# Comprehensive analysis of Transcription Factors identified novel prognostic biomarker in human bladder cancer

**DOI:** 10.7150/jca.58484

**Published:** 2021-07-25

**Authors:** Yihao Liao, Xuanxuan Zou, Keke Wang, Youzhi Wang, Miaomiao Wang, Tao Guo, Boqiang Zhong, Ning Jiang

**Affiliations:** 1 Tianjin Institute of Urology, The Second Hospital of Tianjin Medical University, Tianjin 300211, China.; 2 College of Life Sciences, University of Chinese Academy of Sciences, Beijing 100049, China.

**Keywords:** Transcription Factors, WGCNA, Bladder cancer, Prognosis, Biomarker

## Abstract

**Background:** Transcriptional factors (TFs) are responsible for regulating the transcription of pro-oncogenes and tumor suppressor genes in the process of tumor development. However, the role of these transcription factors in Bladder cancer (BCa) remains unclear. And the main purpose of this research is to explore the possibility of these TFs serving as biomarkers for BCa.

**Methods:** We analyzed the differential expression of TFs in BCa from The Cancer Genome Atlas (TCGA) online database, identified 408 up-regulated TFs and 751down-regulated TFs. We obtained some hub genes via WGCNA model and detected the RNAs level in BCa cells and tissues. Then, the relationship between the expression and clinicopathological parameters was further investigated. Kaplan-Meier curves and the log-rank test were carried out to analyze the relationship between NFATC1, AKNA and five-TFs combination and overall survival (OS). And RT-PCR assay was conducted to further consolidate and verify these results.

**Results:** There were significant differences in the expression of five TFs (CBX7, AKNA, HDAC4, EBF2 and NFATC1) between bladder cancer and normal bladder tissue. In BCa tissue and cell lines, the five TFs were frequently down-regulated, and closely related to poor prognosis. Moreover, the RT-PCR results of five TFs in bladder cancer and normal bladder tissue were consistent with the database results, and reduced TFs could significantly induce or restrain the transcription of many critical factors. The expression level of AKNA and NFATC1 could serve as independent biomarker to predict the overall survival (P<0.05). And the above five TFs combined detection of bladder cancer has higher sensitivity and specificity. Furthermore, differential neutrophils expression between high-risk and low-risk were found, which consolidated the role and function of the five TFs combination model in the progression of BCa.

**Conclusions:** Our analysis effectively provides a newly TFs-associated prognostic model for bladder cancer. The combination of five identified-TFs is an independent prognostic biomarker, which could serve as a more effective therapeutic target for BCa patients.

## Introduction

Bladder cancer (BCa) is the ninth most common malignant tumor worldwide, with high incidence, recrudescence, and mortality rate^1^, ^2^. According to the statistics in America, there were 80470 new diagnosed cases in 2019, including 61700 male patients and 18770 female patients. The death cases due to BCa were 17670, with 12800 males and 4800 females. Based on above statistics, male BCa patients accounted for almost 77% among all BCa cases^3^. Although many advances have been made for the treatment of BCa, such as surgical intervention, adjuvant chemoradiotherapies and radiation therapy, the progression, metastasis, and recurrence rate of BCa remains high. Most BCa patients developed into no signs of significant improvement after a series of treatment^2^, ^4^-^7^. However, the recent research on the genetic characterization of bladder cancer has turned to the molecular therapy. The limited treatment options emphasize the demand for new and effective prognostic biomarkers and new therapeutic modalities^8^.

Transcription factors (TFs) are a large class of chromatin binding proteins, which are associated with different biological processes, including DNA damage repair, transcription process, DNA unwinding and DNA replication^9^. TFs activate or inhibit transcription via binding to DNA helix transactivation or trans-repression domains^10^. Interestingly, the function of TFs is not only related to the process of DNA transcription. Recent studies have identified that TFs play an increasingly important role in human pathology and tumor progression^11^. For example, Vaquerizas proved that almost 164 TFs were tightly associated with 227 different diseases in 2009^12^. Transcriptional disorder is generally considered as a basic feature of tumorigenesis^13^. In BCa, the down-regulation of transcription factor Nrf2, YAP, and c-Myc could inhibit the growth and migration of cisplatin resistant BCa cells^14^. And the imbalance of transcription factors E2F3 and NF-kB may influence the progression and migration of BCa 15. Nevertheless, the function of TFs and their downstream regulatory targets in BCa remain inexplicable.

In present study, we searched the latest TFs list, including 1930 TF genes, and systematically analyzed their transcription profile in TCGA-BLCA cohort to evaluate the potential functions of TFs in BCa. Among them, 408 up-regulated TF genes and 751 down-regulated TF genes were identified. We selected 1159 TFs associated with the progression of BCa, and further analyzed and identified five TFs, including CBX7, HDAC4, EBF2, NFATC1 and AKNA, which were obviously down-regulated in BCa tissues. In particular, the combination of these five TFs is tightly associated with the progress of BCa, which might serve as penitential and critical predictive biomarkers for BCa patients.

## Materials and Methods

### Transcription factor data collection

TRANSFAC (http://generegulation.com/pub/databases.html), CISBP (http://cisbp.ccbr.utoronto.ca/), TRRUST (https://www.grnpedia.org/trrust/) are the most common transcription factor databases during bioinformatics analysis. We collected and classified these transcription factors from these databases, and removed these duplicate TFs from each database, then obtained a confederate TF data set (including 1930 TFs, 1889 of which have expression profile data). Bladder cancer dataset in TCGA downloaded and the differentially expressed TFs (DE-TFs) were screened. The mRNA expression profile data and clinical information data of bladder cancer (BLCA) were downloaded from TCGA database (https://www.cancer.gov/about-nci/organization/ccg/research/structural-genomics/tcga), and the differential expression between normal tissues and bladder cancer tissues was analyzed by R's DESeq2 software package. The TFs data set was combined to screen out the differential expressed TFs between normal tissue and BCa tissue (|log2FoldChange|>1 & padj<0.05; 408 up-regulated TFs, and 751 down-regulated TFs).

### Weighted gene co-expression network analysis and hub gene extraction

Weighted correlation network analysis (WGCNA) is a system biology method to describe gene association patterns between different samples. It is usually used to identify highly coordinated gene sets, and identify candidate biomarker genes or therapeutic targets according to the interconnectivity of gene sets and phenotypes. The construction of WGCNA network mainly includes following steps: firstly, Pearson correlation coefficient (indicating the synergistic effect of gene expression) is calculated through the gene expression profile. Secondly, in order to avoid the hard classification of correlation coefficients, the soft threshold recording method (the weighting function used to concatenate the elements in the adjacency matrix) is used in WGCNA. In this paper, power function is used to enhance the strong correlation network, weaken the correlation network, continuously calculate the elements, and select the most appreciate β value through screening to obtain the network more conforming with the scale-free network model. Thirdly, in order to further consider the relevance of biological significance instead of expression, the topological overlap matrix (TOM) is constructed to better simulate the network model. The idea of the model demonstrates that the relationship between two genes could be affected by the interaction of intermediate genes, which describes the similarity of gene expression profiles more accurately. Finally, in order to facilitate the construction of the modules, a dissimilarity matrix needs to be defined, which can be obtained by subtracting the topological overlap matrix from 1 (1-TOM). The dissimilarity matrix is used to cluster the matrix via the hierarchical clustering method, and the dynamic cutting tree method is used to identify the modules to get different modules. The correlation analysis of cancer and normal tissue was analyzed through the topological overlap matrix, and the modules with strong correlation with cancer traits were obtained. These modules may contain important transcription factors involved in the occurrence and development of cancer.

### GO analysis and KEGG analysis

In order to explore the function of these genes in the modules strongly related to cancer traits or signaling pathways screened by WGCNA, next GO analysis and KEGG analysis of these extracted genes were performed through the cluster Profiler package in R. GO analysis could be mainly divided into three categories: Molecular Function (MF), Biological Process (BP), and Cellular Components (CC). KEGG analysis could reveal the signal pathways processes involved in these genes.

### Cox regression model construction

These genes and their expression profiles of several modules screened by WGCNA analysis were extracted. First, all the genes significantly related to prognosis were screened by TCGA-BLCA univariate Cox regression analysis via survival and survminer in R (p<0.05). Then multivariate Cox regression analysis was performed with R package to select prognostic-related markers to construct a prognostic survival model. Finally, the prognostic risk model was constructed for the genes related to prognosis, and the contribution coefficient of each gene to the risk was obtained, which is employed to establish a comprehensive risk prediction model.

Prognostic risk score:


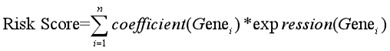


Among them, Genei represents the i-th prognostic gene; expression (Genei) represents the expression level of the patient's i-th prognostic gene; coefficient (Genei) is the coefficient of each prognostic gene.

### Prognostic model evaluation and data set validation

In order to verify the effectiveness of the model via TCGA-BLCA data, the risks are divided into two groups according to the risk value of the case in TCGA-BLCA data. And the relationship between the risk value and prognosis was discussed. At the same time, we used external data (METABRIC) to validate the model, and the ROC curve was drawn to prove its effectiveness. The survival curve was drawn using the survival package in R, while the ROC curve was drawn via survival ROC package. In addition, combined with the clinical situation of patients, the heat map of model-related genes in high-risk and low-risk groups was used to reveal the difference of gene expression between high-risk group and low-risk group and different clinical states.

### CIBERSORT Immune Infiltration Difference Analysis

The same tumor type may have different immune activity between different individuals due to the high heterogeneity of tumor tissues. Therefore, different samples may be in different immune microenvironment. The transcriptional expression level of tumor samples could reflect the composition of various immune-related components, CIBERSORT can extract the composition of 22 kinds of immune infiltration-related cells by analyzing the tissue expression profile. Using this data, we can further infer the immune infiltration status of different cancer tissues. The analysis process is implemented using the R package analysis provided by the CIBERSORT official website.

### Patients and tissue specimens

This research collected 10 BCa and matched para-cancerous tissue from BCa patients who underwent surgical resection without any neoadjuvant chemotherapy and radiotherapy in the Department of Urology, the Second Affiliated Hospital of Tianjin Medical University (China) from 2017 to 2019. The inclusion criteria: these patients underwent total cystectomy; and the exclusion criteria: these patients underwent electrocystectomy and puncture. This study was approved by the Institutional Review Committee of this hospital.

### Cell lines acquisition and transfection

Four bladder cancer cell lines (T24, 5637, 87, EJ) and human immortalized uroepithelial cell line SV-HUC-1 were purchased from American Type Culture Collection (ATCC). And human BCa cell lines were cultured in RPMI-1640 medium containing 10% FBS and 1% penicillin-streptomycin at 37℃ with 5%CO_2_. While the SV-HUC-1 cell line was cultured in F-12k Nutrient Mixture containing 10% FBS and 1% penicillin-streptomycin at above conditions. We selected BCa T24 cell for subsequent mechanism research, and transfected T24 cell with designed corresponding siRNA of HDAC4, NFATC1 and CBX7 according to lipo2000 transfection protocol. We extracted total RNA after 48h transfection and performed PCR for subsequent research.

### RNA extraction and qRT-PCR

Total RNA was extracted from BCa tissue, para-cancer tissue, BCa cell lines and SV-HUC-1 cell using traditional TRIzol reagent, and cDNA was synthesized by cDNA Synthesis SuperMix (gDNA Purge) (Novoprotein). The quantitative real-time polymerase chain reaction (qPCR) was executed by SYBR Premix Ex Taq II assays, and GAPDH was used as an internal reference for normalization. The relative expression ratio of these genes was calculated through 2^-ΔΔCq^ method. These related primer sequences and siRNA sequences are showed in Table S1.

### Statistical analysis

Statistical analyses were performed by GraphPad Prism 5.3 and software SPSS 21.0. All data was presented as the mean ± standard deviation of at least three independent experiments by different experimenters. T-test and Chi-square test were used for data analysis. P<0.05 was considered to bet a statistically significant difference.

## Results

### Transcription factor are differentially expressed in bladder cancer

**Figure 1:**
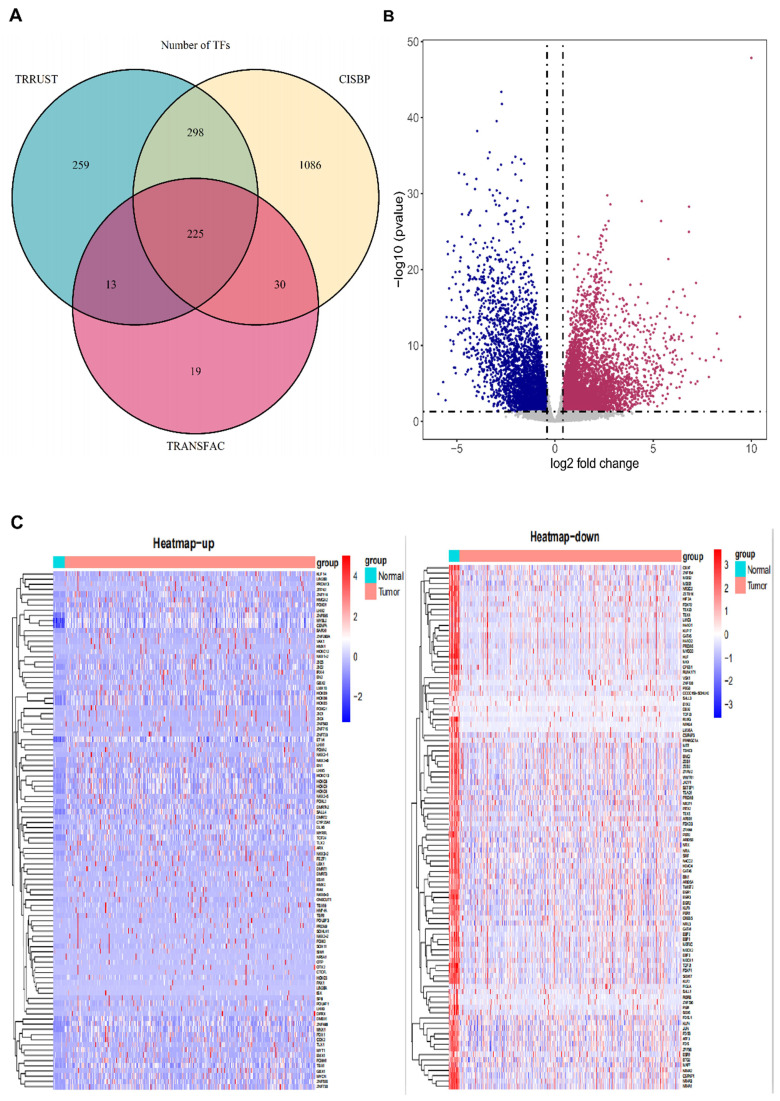
** Transcription factor are differentially expressed in bladder cancer.** (A) The Venn plots depict the sources of the 1930 TFs. (B) The volcano plot depicts the differential expression of all genes in BCa from TCGA database. (C) The heatmaps display the top-100 up-regulated (left panel) and top-100 down-regulated (right panel) TFs in BCa.

We obtained 1930 transcription factors via searching in TRANSFAC (http://generegulation.com/pub/databases.html), CISBP (http://cisbp.ccbr.utoronto.ca/) and RRUST (https://www.grnpedia.org/trrust/) cohort, and the Venn plot presented the specific origin of the 1930 TFs (Fig. 1A). Next, we searched the differentially expressed genes of BCa expression profile in the TCGA database (https://www.cancer.gov/about-nci/organization/ccg/research/structural-genomics/tcga), the results were presented in the volcano plots (Fig. 1B). The specific list of transcription factors and P value were displayed in Table S2. And the relative expression profile of 1930 TFs were found in the TCGA database to explore the abnormal expression level of TFs and new prognostic biomarkers in BCa development. TFs differential expression analysis was performed by 401 paired BCa tissue and normal bladder tissue. The results showed that 408 TFs were up-regulated and 751 TFs were significantly low expressed in BCa tissue. These top-100 up-regulated and top-100 down-regulated TFs identified in BCa were displayed in the heatmaps (Fig. 1C). The detailed list of dysregulated transcription factors and relative P values were obtained in the Table S3 (down-regulated) and S4 (up-regulated).

### Construction of BCa co-expression modules

**Figure 2:**
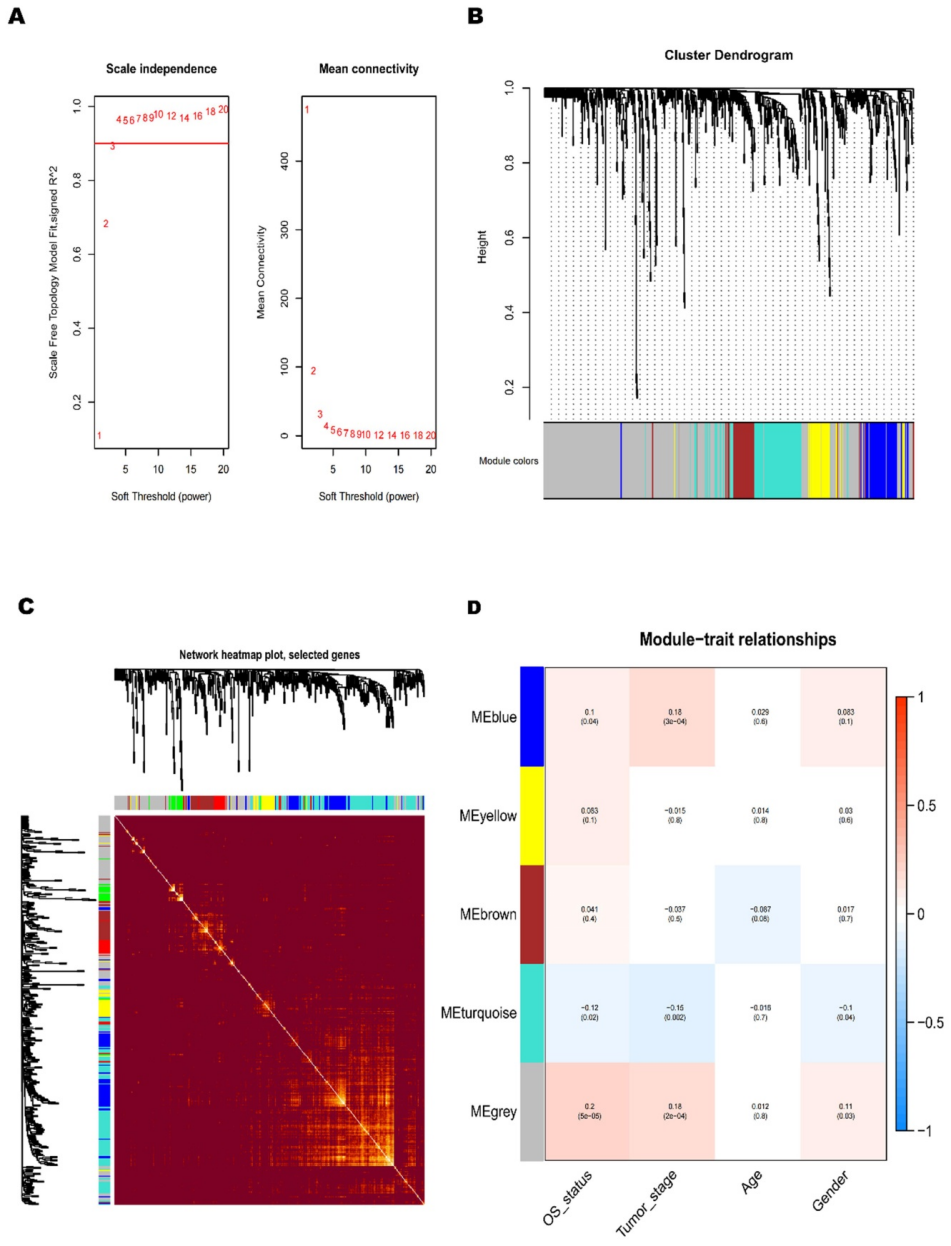
** Construction of BCa co-expression modules.** (A) Analysis of network topology for various soft-threshold powers. (B) The clustering dendrograms of genes is based on dissimilarity of topological overlap, and the assigned module colors. (C) The heatmap depicts the topological overlap matrix (TOM) among genes based on co-expression modules. (D) Analysis of module-trait relationships of BCa is based on TCGA online database (Each row corresponds to a module eigengene, and each column corresponds to a trait).

To further understand the dysfunctional TFs, we tried to construct a WGCNA network. The independence and average connectivity degree of co-expression modules were decided by power value (β) and scale R^2^ value. First, we plotted a set of soft-thresholding powers, and the scale R2 reached 0.90 when the power value was equal to 4 (Fig. 2A). Through soft thresholding with β=4, we defined the adjacency matrix to construct and identify the differential co-expression TFs modules in BCa samples. A cluster dendrogram of 1159 chosen TFs were constructed based on the TOM-based dissimilarity measure. These co-expression modules identified by cluster analysis were allocated in different colors (Fig. 2B). The interactions of these co-expression modules were analyzed with the Pearson correlation coefficient (Fig. 2C). The darker background indicated a higher module correlation.

### The relationship between TFs co-expression modules and clinicopathological parameters

In the principal component analysis of each module, the first principal component was selected as its characteristic TFs. Other clinicopathological parameters, including gender, age, tumor stage and OS-associated status, were related to these different co-expression modules, and the strongest relevant blue module was identified (Fig. 2D). A heatmap of the correlation between module characteristic TF and clinicopathological parameters of BCa presented the correlation coefficient (R) and significant difference (p value). And the Module- parameters diagram describes 174 TFs in Blue module, 78 TFs in Yellow module, 93 TFs in Brown module, 166 TFs in Tuiquoise module and 648 TFs in Grey module (Table 1). And the TFs of Blue, Yellow Brown and Tuiquoise module were selected for further analysis, while the TFs of Grey module did not meet the criteria and were excluded from the analysis. The key TFs of each module may become the candidate biomarkers for the certain clinicopathological parameters. The correction analysis of TFs module and clinicopathological parameters presented that the co-expression grey module was obviously related to OS-status (R=0.2, P<0.0001), and the co-expression of blue module was tightly associated with tumor-stage (R=0.18, P<0.001). The correlation results in the blue module demonstrated that the TFs were significantly associated with tumor stage, implying that the TFs might provide a potential biomarker for the prognosis of BCa. Considering the relationship between the module and clinicopathological parameters, we selected the co-expression blue module for next analysis.

### Functional enrichment gene analysis of co-expression blue module

**Figure 3:**
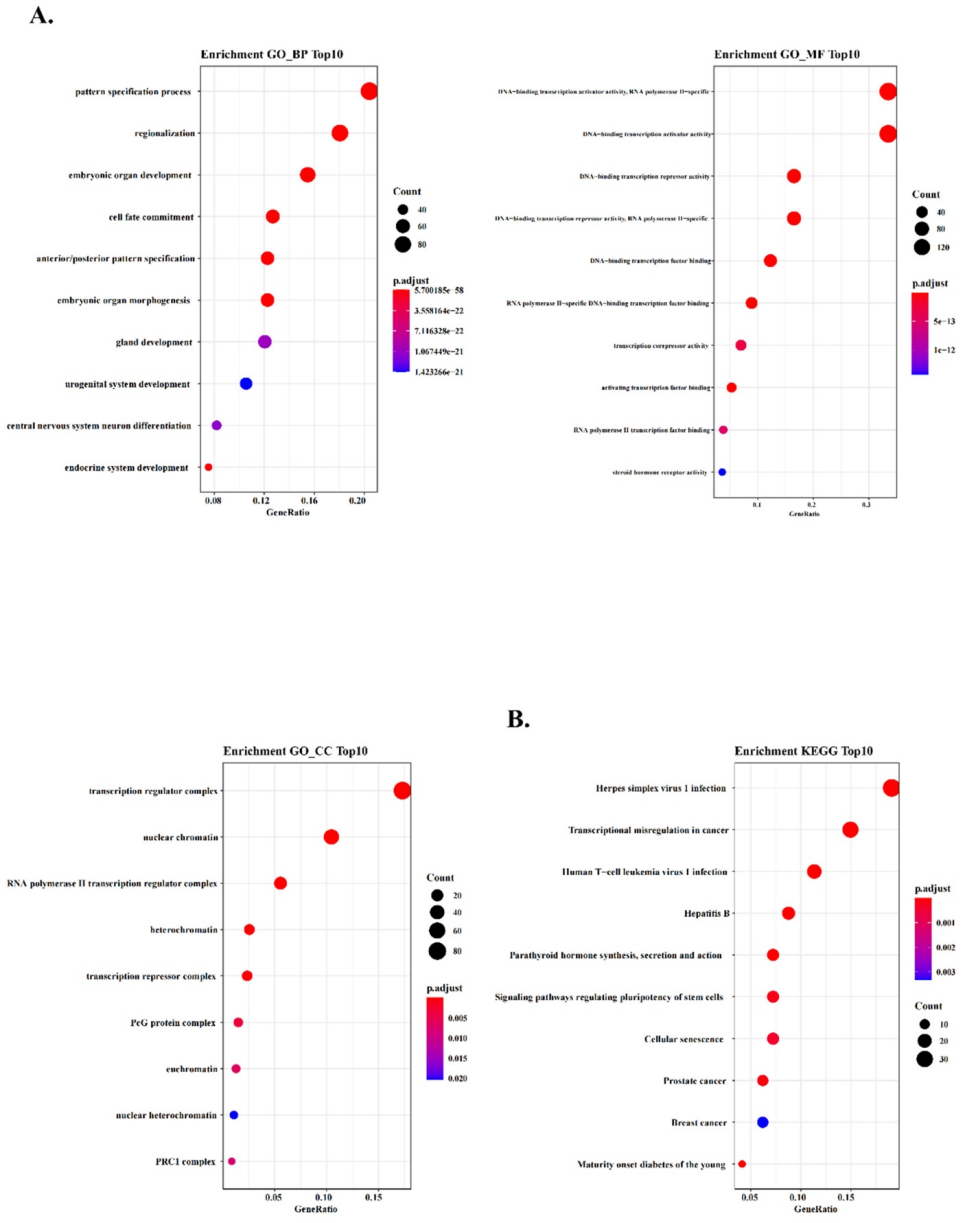
** WGCNA predicts GO and KEGG pathways associated with the five‐TFs signature.** (A) The GO analysis of the co‐expressed genes is shown in blue module (GO-BP; GO-MF and GO-CC). (B) The KEGG analysis of the co‐expressed genes is shown in blue module.

According to the heatmap above, we selected all 174 TFs of the blue module and the hub TFs (containing CBX7, HDAC4, EBF2, NFATC1 and AKNA). To further explore the potential function of these TFs in co-expression blue module, we conducted Gene Ontology (GO) analysis to analyze the key relative function. The results of enrichment GO_BP demonstrated that these TFs were associated with many critical life processes, such as morphogenesis of many important organs and development of urogenital system. These TFs might play a formidable role in variety of cells proliferation, and their dysregulation might cause the occurrence of urogenital tumors (Fig. 3A). And we performed GO_MF enrichment analysis and found that these TFs were significantly associated with the activity of DNA binding transcription activators and DNA binding transcription receptors (Fig. 3A). In addition, GO_CC enrichment analysis demonstrated that transcription regulatory complex and nuclear chromatin were tightly associated with the function of these TFs, suggesting that these TFs might regulate tumor formation and progression (Fig. 3A). To further ascertain the function of these TFs, we found that transcriptional dysregulation in cancer was enriched by Kyoto Encyclopedia of Genes and Genomes (KEGG) pathway analysis (Fig. 3B). The results implied that these TFs might regulate many cancer-related factors and tumor development by regulating the process and status of transcription.

### Five-TFs combination is a novel prognostic biomarker for BCa and associated overall survival

**Figure 4:**
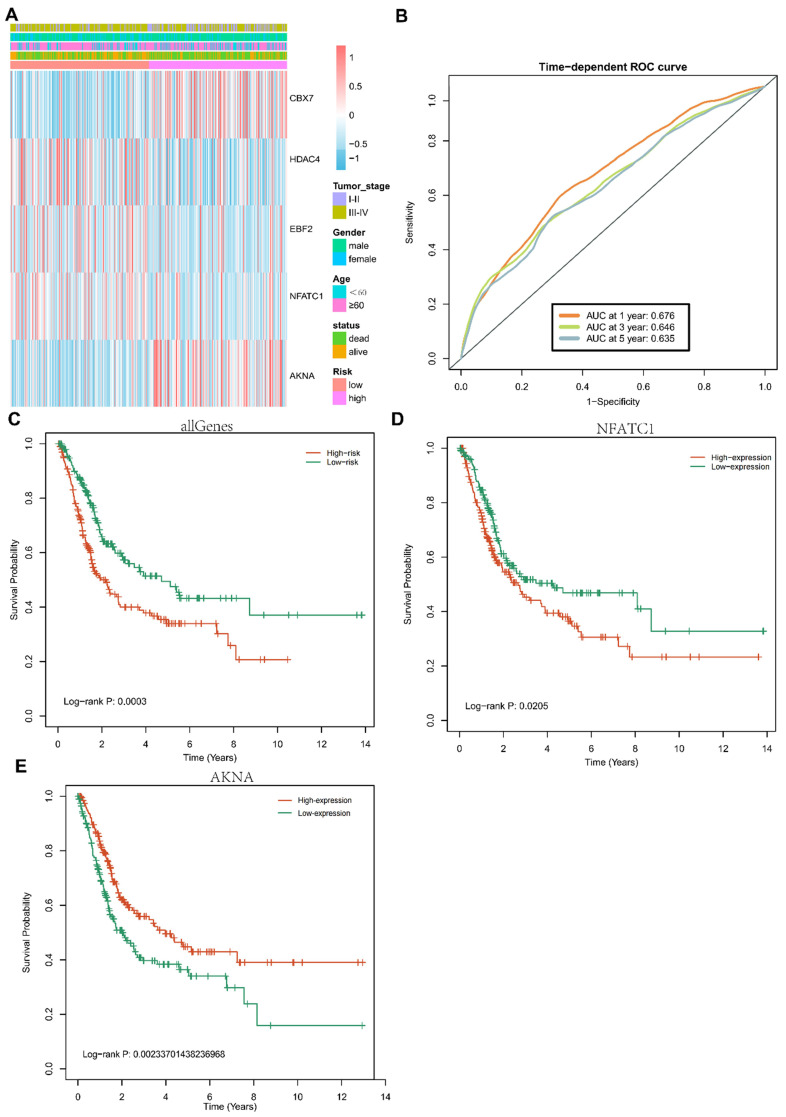
** Prognostic value of the five TFs combination and associated with clinical outcome of bladder cancer.** (A) Hierarchical clustering heatmap and dendrogram of bladder cancer samples based on the expression patterns of the five selected TFs. (B) Receiver operating characteristic (ROC) analysis of the risk scores for overall survival prediction in the TCGA dataset. (C) Kaplan-Meier survival curves for bladder cancer samples classified into high-risk and low-risk groups using the five TFs combination signature. P-values were calculated using the log-rank test (P=0.0003). (D) Kaplan-Meier survival curves for bladder cancer samples classified into high-expression and low-expression groups based on NFATC1, P-values were calculated using the log-rank test (P=0.0205). (E) Kaplan-Meier survival curves for bladder cancer samples classified into high-expression and low-expression groups based on ANKA, P-values were calculated using the log-rank test (P=0.00233).

Univariate cox regression and multivariate cox regression were conducted in these TFs of Blue models. Five TFs, including CBX7, HDAC4, EBF2, NFATC1 and AKNA, were identified as prognostic biomarkers of BCa. To investigate the specific relationship among these five TFs and the clinicopathological parameters (including tumor stage, gender, OS-status and age), we divided these BCa patients into two groups based on high risk and low risk, cox proportional hazards regression model was used for multivariate analysis. The expression of five-TFs combination models was compared with other clinical parameters as covariates to explore whether the expression of five-TFs combination model was an independent prognostic factor in BCa. The analysis results were presented in the heatmap (Fig. 4A). These patients with BCa were divided into two groups according to high risk and low risk, the results demonstrated that the five-TFs combination had a great prediction on the progression from stage I&II to stage III&IV (Table 2; P<0.01). The results presented that the five-TFs combination might serve as a potential prognostic biomarker for BCa. Next, we explored the effects of five-TFs on predicting 1 year, 3 years and 5 years survival. The results were presented in the ROC curve (Fig. 4B), which could evaluate the prognostic efficiency in patients with BCa. We found that the AUC was 0.675 at 1 year, 0.645 at 3 years and 0.635 at 5 years. Kaplan-Meier Survival analysis showed that patients with high risk in five-TFs combination model presented poor overall survival (P<0.05; Fig. 4C), and these BCa patients with high expression of NFATC1 and low expression of AKNA were tightly associated with poor OS (P<0.05; Fig. 4D,E). The above results showed that the five-TFs combination model could serve as a candidate prognostic biomarker for BCa patients.

### The differential expression of neutrophils was associated with the risks of five-TFs combination

**Figure 5:**
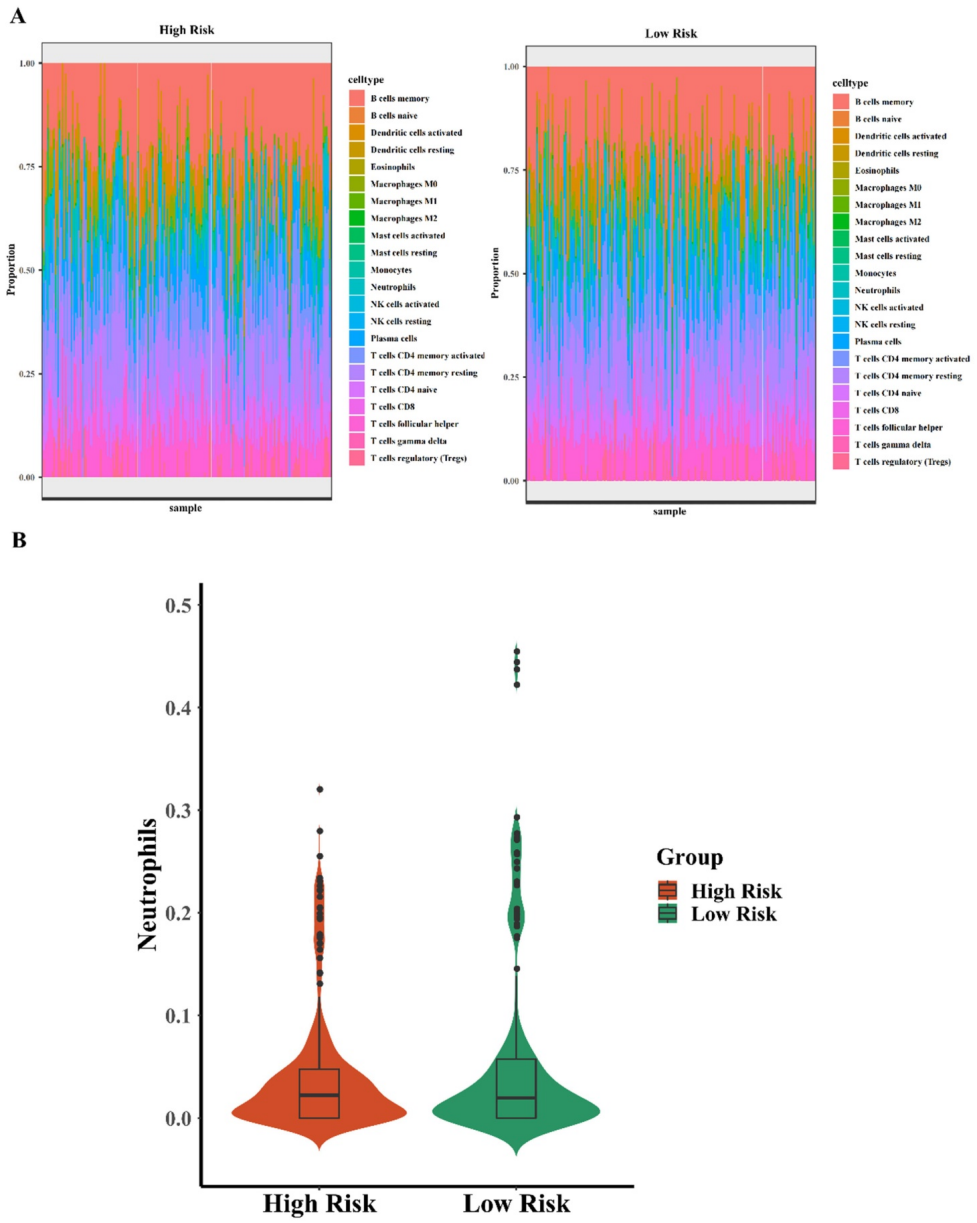
** The differential expression of neutrophils is associated with five-TFs combination.** (A) The differential expression analysis of 22 immune cells based on high risk and low risk of the five TFs combination signature (left panel: high risk, right panel: low risk). (B) The violin illustration depicts the differential expression of neutrophils based on high risk and low risk of the five TFs combination characteristics.

It has been known that immune infiltration was frequently found in the early anti-cancer defense, which can arrive in where the tumor cells grow abnormally. It reminds that related immune cells early infiltration may predict the tumorigenesis and carcinogenesis, lots of researchers have found the relationship between immune infiltration and carcinogenesis. For example, Xiaoyan Fan et al. identified that TACC3 is a prognostic biomarker for kidney renal clear cell carcinoma and correlates with immune cell infiltration and T cell exhaustion^16^. Hai Zhu et al. found that ITGA5 is a prognostic biomarker and correlated with immune infiltration in gastrointestinal tumors^17^. Moreover, Hanji Huang et al. identified that C1Q is a critical prognostic factor and related with immune infiltrates in osteosarcoma^18^. There are massive evidences for supporting the role of immune infiltrates in the processes of carcinogenesis and development. In this study, we tried to explore the relationship between immune infiltrates and the high risk and low risk of the five-TFs combinations in BCa, further explored the efficiency of the five-TFs combinations predicting the changes of related immune cells. To analyzed the effect and potential function of five-TFs combination model, we explored the differential expression of common 22 immune-associated cells (such as B lymphocytes and neutrophils) in high risk and low risk with the five-TFs combination model. The analysis results were shown in the heatmap (Fig. 5A). We found that high risk BCa patients with five-TFs combination model presented lower expression level of neutrophils compared with these low risk patients (Fig. 5B; P<0.05), while other immune cells have no obvious discrepancy between two groups(P>0.05). It has been known that neutrophils are the most common immune cell in anti-inflammatory response and natural anti-tumor response processes, and elevated neutrophils usually implied that abnormal cell growth or pathogen infection. In previous study, Chang Cui et al. found that neutrophil elastase selectively killed cancer cells and attenuated tumorigenesis^19^. Neutrophil-secreted elastase shows dramatic selectivity, inducing cell death in tumors and at metastatic sites in animal models while sparing proximal healthy cells, which further implied that neutrophil was a critical factor for the development and treatment of cancers. In the early stage of BCa occurrence, lots of neutrophils will move and gather into the area of abnormal growth, and some researchers thought that elevated neutrophils might serve a signal of tumor occurrence or abnormal growth. We found that these BCa patients with high risk almost have higher neutrophils expression, which implied that the five-TFs combination model might be a perfect biomarker for early immune infiltration in bladder cancer patients. And neutrophils infiltration might further consolidate the accuracy of the TFs model for BCa diagnosis. These results suggested that the five-TFs combination model could serve as a potential role in predicting immune infiltration and inflammation associated processes. Other immune cells have no obvious changes between high and low risk of the TFs model, but the function of which still are critical for the whole anti-tumor processes. The complete predicting function of the TFs model needs further analysis and research. The results showed that these patients with high risk of the five-TFs combination have higher neutrophils expression, while other immune cells have no obvious differences. We could further predict BCa prognosis and the anti-tumor effects of neutrophils based on the five-TFs combination model risk. And the expression of neutrophils means that the percentage of neutrophils among all immune cell infiltration, we could predict the prognosis of BCa and the intensity of neutrophils immune infiltration via TFs mRNA expression and model risks, which implied that we can predict immune infiltration and carcinogenesis based on high risk and low risk of the five -TFs combination model. And the specific mechanism and processes need further research and analysis.

### Protein-protein interaction (PPI) and a transcription factor network analysis with five-TFs combination

**Figure 6:**
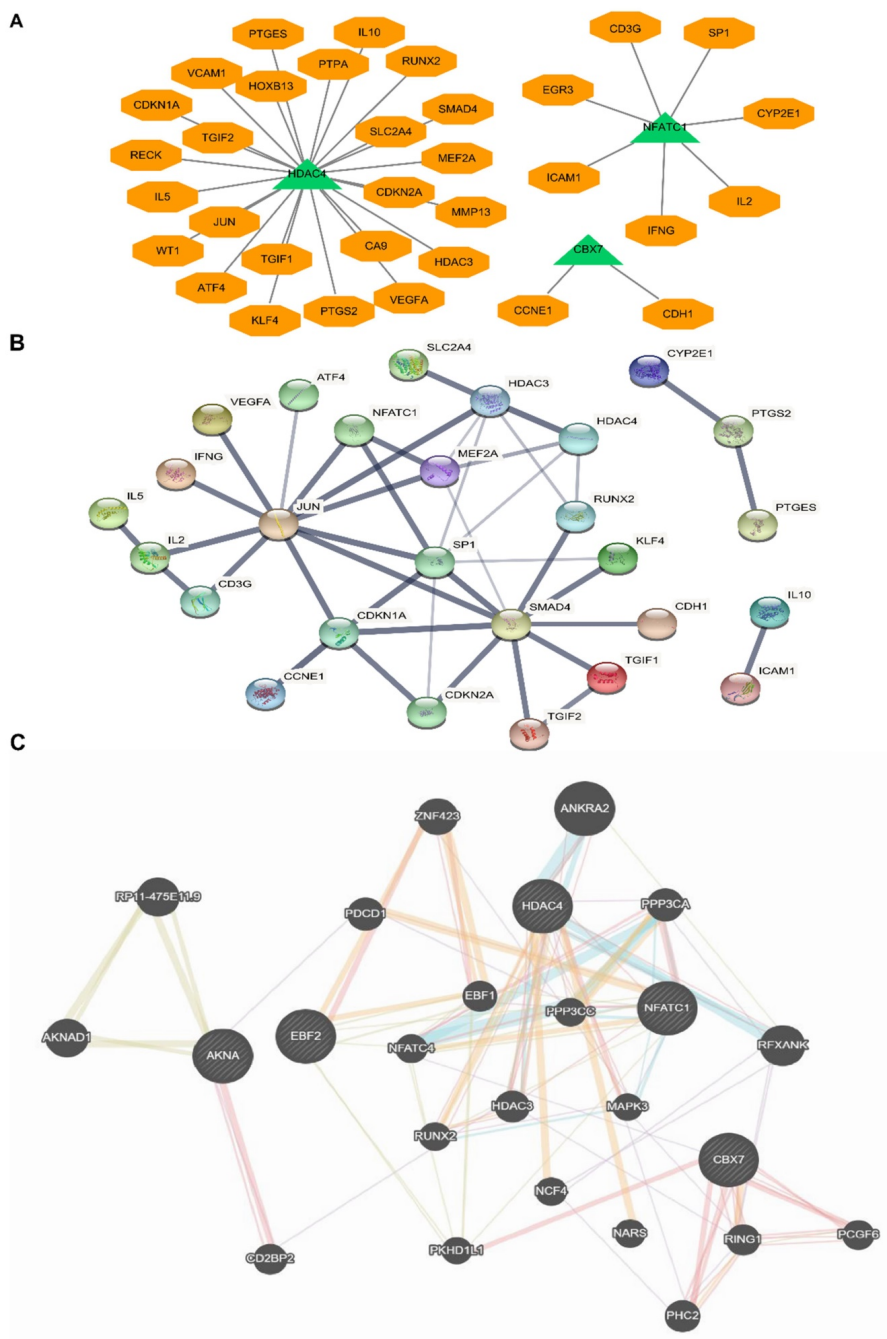
** Protein-protein interaction (PPI) network analysis and a transcription factor network analysis with five-TFs combination.** (A) The potential binding sites or targets of the five TFs were predicted based on the same transcription direction. (B) Protein-protein interaction (PPI) network analysis based on the five TFs combination was established. (C) The comprehensive network was analyzed via genemania online analysis database.

Due to the above five TFs were candidate biomarkers for BCa patients, we further researched whether these TFs could influence some important tumor-associated signaling pathway proteins or factors, and analyzed the complex network of these five TFs. Many critical protein and factors were found to be directly and tightly associated with these TFs (Fig. 6A). For example, we found that JUN, IL-10, IL-5 and SMAD4 were closely related to HDAC4, NFACT1 and IL-2, and CBX7 and CDH1 were also connected with each other. To further identify the factors interacted with the above five TFs, we uploaded the five TFs to STRING for protein-protein interaction (PPI) analysis (Fig. 6B). Finally, to further consolidate the above results, we analyzed the sophisticated interaction network via gene mania online analysis database, and the results demonstrated that the five TFs related to each other and regulated by many critical proteins (Fig. 6C). These results indicated that the interaction effects between proteins were not only expressed at the expression level, but also signaling cascade. All above results implied that the five TFs were crucial for the occurrence and development of bladder cancer via lots of critical cancer related pathways, however, the specific mechanism still needs further research. This study preliminary promoted the hypothesis that the five critical TFs regulated BCa processes of occurrence and development by influencing the transcriptions of these related factors, which will be the foundation of subsequent researches.

### The five-TFs combination is an independent prognostic biomarker

**Figure 7:**
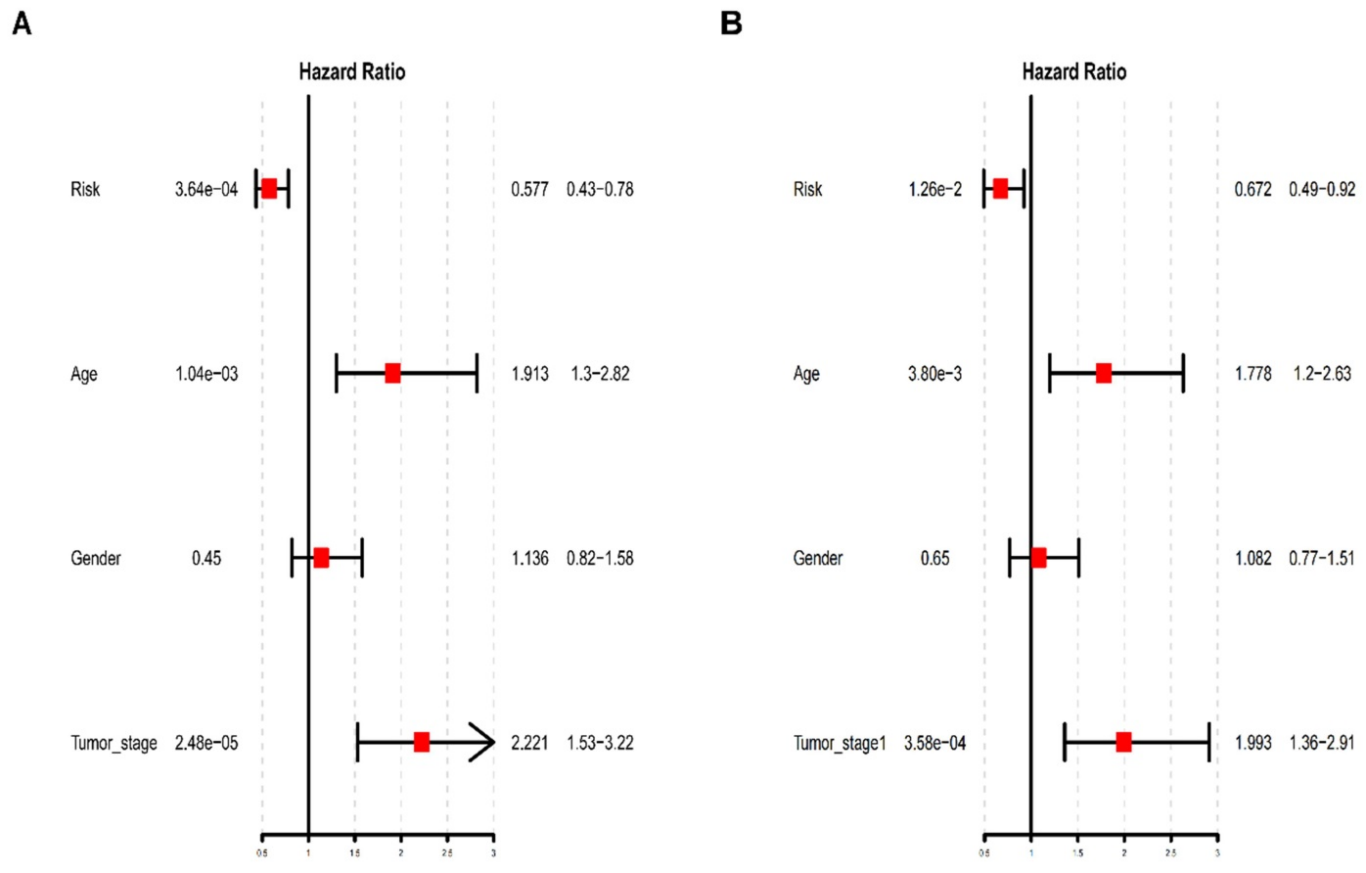
** Univariate and multivariate analysis of clinicopathological factors and the five-TFs combination in BCa malignant progression.** (A)Univariate analysis of clinicopathological factors and the five-TFs combination in BCa malignant progression. (B) Multivariate analysis of clinicopathological factors and the five-TFs combination in BCa malignant progression.

To further determine whether the risk prediction model of the five-TFs combination is an independent predictive biomarker of survival in BCa patients, univariate and multivariate analyses were used to assess the prognostic association between some known clinicopathological risk factors for BCa progression and the newly identified five-TFs combination. The above mentioned clinicopathological risk factors, including age, gender and tumor stage, were considered. As we expected, besides of gender, the prognostic efficiency of the five-TFs combination was similar to these of age and tumor stage (P<0.01; Table 3; Fig. 7A). Another multivariate analysis further revealed that the five-TFs combination remained an independent prognostic risk factor for the survival of BCa patient (P= 0.012; Table 3; Fig. 7B).

### The mRNA expression level of five-TFs in BCa and normal bladder tissue

**Figure 8:**
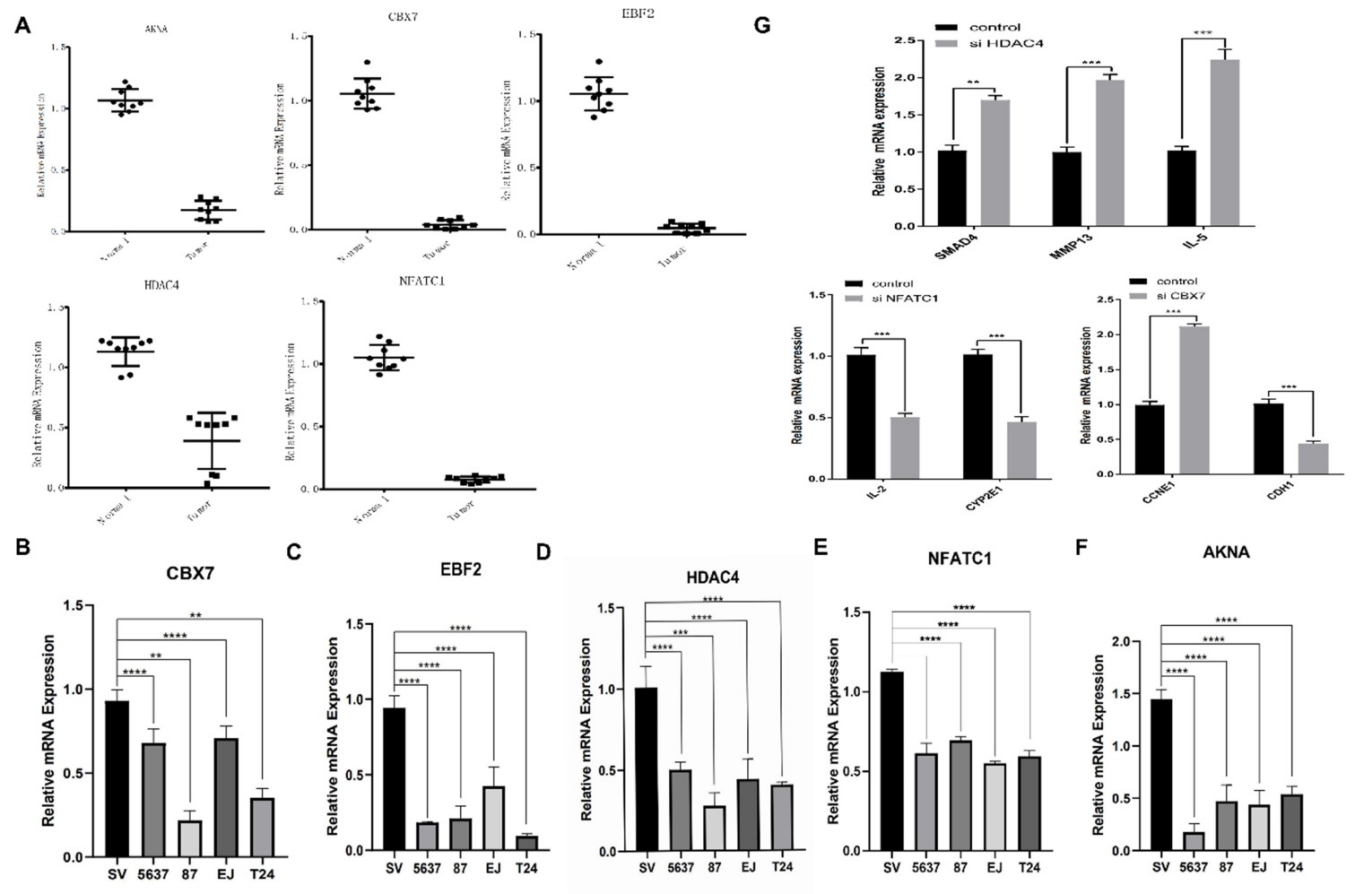
** qRT-PCR validation of the five identified transcription factors in BCa cell models and bladder cancer tissue.** (A) The mRNA expression level of the five TFs in bladder cancer tissue compared with normal tissue. (B-F) The mRNA expression levels of the five TFs in bladder mucosal immortalized cells SV compared with BCa cells T24, 5637, 87 EJ. (G) Some critical cancer related factors (SMAD4, MMP13, IL-5, IL-2, CYP2E1, CCNE1 and CDH1) mRNA expression in bladder cancer T24 cell transfected with si HDAC4, si NFATC1 and si CBX7.

To further verify the above analysis results from TCGA online database, we performed qRT-PCR to validate the relative mRNA expression level of five TFs (including CBX7, HDAC4, EBF2, NFATC1 and AKNA) in BCa tissue and normal bladder tissue, which could predict BCa and associate with survival. The results demonstrated that there was obviously differential expression of five TFs between BCa tissue and normal bladder tissue, and the expression of these five TFs in the normal bladder tissue was significantly higher than that in BCa tissue (Fig. 8A). These results were consistent with the expression which we analyzed from the database. And next, another qRT-PCR was performed to dig out the differential expression of the five TFs among one control bladder immortalized cell (SV-HUC) and several BCa cells (including T24, 5637, 87 and EJ). We found that the expression levels of these TFs were lower in these BCa cell lines than that in the control bladder immortalized cell (Fig. 8B-F). The above results demonstrated that the expression of these TFs was down regulated in BCa compared with these patients without bladder cancer, which implied it was feasible that observing the expression of the five TFs predicts the tumorigenesis. Previous results showed that the five TFs might be involved in many critical factors and pathways, such as SMAD4, MMP13, IL-5, IL-2, CYP2E1, CCNE1 and CDH1 and so on. To further verify our hypothesis, we transfected bladder cancer T24 cell with designed si HDAC4, si NFATC1 and si CBX7. The results demonstrated that reduced HDAC4 could significantly induce the transcription and expression of SMAD4, MMP13 and IL-5; and reduced NFATC1 could obviously restrain the transcription and expression of IL-2 and CYP2E1, declined CBX7 significantly induce the transcription of CCNE1 and restrain the expression of CDH1 (Fig. 8G). All above results demonstrated that the five TFs were important for the transcription and expression of various cancer related factors, which further consolidated the role of the five TFs combination in the occurrence and development of bladder cancer. However, the limitations of this study were obvious, we only detected the expression of TFs in BCa tissue and cells, which was not convenient for current clinical practice. We will try to detect the expression of TFs in blood or urine in next experiments, which may promote greatly the prognostic efficiency of BCa. If the results of blood or urine were consistent with that of BCa tissue and cells, we believe that detecting the expression of five TFs will become a new milestone for the history of BCa diagnose and treatment, which will bring huge improvement for lots of BCa patients.

## Discussion

Recently, more and more studies have revealed the core regulatory roles of TFs during the progression and evolution of various cancers^20^. By regulating the transcription of cancer-related factors or pathways, TFs can induce carcinogenesis, promote cancer progression or restrain the function of tumor suppressor genes. However, the specific clinical significance and relative function of TFs in BCa remains unknown. This research systematically analyzed the related disordered TFs in BCa and preliminarily identified the five-TFs combination network (CBX7, HDAC4, EBF2, NFATC1 and AKNA) as important prognostic-related biomarker, which was associated with BCa progression. The results demonstrated that the expression of these five TFs was down-regulated in BCa patients, and the overall survival rate of high risk patients with BCa in the five-TFs combination model was poor (P<0.01). The five-TFs combination model may play a critical prognostic role of BCa. In this study, five-co-expression TFs modules were constructed by 1930 TFs from 401 human BCa clinical samples. The samples were provided by WGCNA, to determined critical modules tightly associated with some important clinicopathological characteristics. WGCNA was conducted to filtrate these co-expression TFs clusters to determine prognostic biomarkers of BCa. In this research, PPI network analysis and WGCNA were performed to analyze the critical TFs related to the progression of BCa, to explore the potential mechanisms, and to further identify effective prognostic biomarkers of BCa. Survival analysis of these identified TFs presented that the high risk of the five-TFs combination model was associated with poor overall survival. Chong Shen et al. identified a potential prognostic biomarker, LncRNA ENST00000598996 and ENST00000524265, by WGCNA and PPI network analysis of BCa^8^. And previous studies have reported that lncRNA XIST^21^, MAGI2‑AS3^22^ and ADAMTS9‑AS2^23^ are prognostic biomarkers of these patients with BCa and plays a critical role in BCa progression via various tumor-related pathways. Furthermore, the TFs network analysis of the specific mechanism in BCa was performed. These TFs have been reported in previous researches and are tightly related to many critical biological processes. CBX7, Chromobox 7, is the component of Polycystic group (PcG), multiprotein PRC1-like complex, which is necessary to maintain the transcriptional repression of many genes (including Hox genes) tin the whole development process^24^. It is associated with many diseases, including melanoma^25^, ^26^ and ovarian clear cell adenocarcinoma^27^. HDAC4, Histone Deacetylase 4, is responsible for the deacetylation of N-terminal lysine residues of the core histones (H2A, H2B, H3 and H4)^28^. And this process provides a marker for epigenetic repression and plays an important role in transcriptional regulation, cell cycle progression and developmental events. The dysregulation of HDAC4 is associated with many diseases, such as vascular inflammation^29^ and brachydactyly^30^, ^31^. EBF2, EBF Transcription Factor 2, is a non-basic, helix-loop-helix transcription factors belongs to the COE (Collier/Olf/EBF) family, has a well conserved DNA binding domain and plays a critical role in regulating osteoclast differentiation. Many diseases are related to abnormal regulation of EBF2, such as Kawasaki Disease^32^, Inguinal Hernia^33^ and Kallmann Syndrome^34^, ^35^. NFATC1, Nuclear Factor of Activated T Cells 1, plays a role in the inducible expression of cytokine genes in T-cells (especially in the induction of the IL-2 or IL-4 gene transcription) and regulates many important genes of osteoclast differentiation and function. NFATC1 associated diseases include Cherubism^36^-^38^ and Osteonecrosis^39^, ^40^. AKNA, AT-Hook Transcription Factor, regulates microtubule tissue and specifically activates the expression of CD40 receptor and its ligand CD40L/CD154(two cell surface molecules on lymphocytes, which are critical for the development of antigen-dependent-B-cell). It remains unclear how it can act as both a transcription factor and a microtubule organizer, and more evidence is required to explain these two apparently contradictory functions^41^, ^42^. These TFs were frequently downregulated in BCa tissue compared with para-cancerous tissue from the online database, suggesting that these TFs may be involved in the pathogenesis of the disease. Additionally, our research proved that these BCa patients with high risk of these five TFs combination had a worse overall survival rate. CBX7 could inhibit the invasion ability of BCa 26, and Kaletsch A et al. identified that HDAC4 served as a tumor promoting factor, and Histone deacetylase inhibitor (HDACi) was considered to be a promising anti-cancer drug, which could be employed for the treatment of urothelial carcinoma (UC) 43. Kawahara T et al. found that NFATC1 was a potential prognostic actor^44^ and a critical tumor promoting factor to promote the progression of BCa^45^. The other identified TFs have not been reported in previous study, therefore our efforts add a valuable supplement to identify potential transcription factor related biomarkers for BCa patients. And transcription factor has been known to be the critical regulator of many important processes and protein, and the dysregulation of TFs usually cause the abnormal expression of proto-oncogenes or tumor suppressor genes. The dysregulation of TFs almost is the first step of the occurrence of various cancers. In this study, we found that the five-TFs combinations were potential prognostic biomarker and associated with the overall survival for BCa, and we can predict the occurrence of BCa by observing the expression of the five TFs. The results of PPI demonstrated that the five TFs were tightly associated with many critical cancer related factors or pathways, such as SMAD4, MMP13, IL-5, IL-2, CYP2E1, CCNE1 and CDH1. We further found that reduced TFs significantly induced or restrained the transcription or expression of these factors, which implied that the five TFs might control the progression of BCa via mediating with these factors. All above results further consolidated the critical role of the five TFs on the occurrence and development of BCa. We will continue to explore the deep mechanism of five TFs influencing the development, and restraining the expression of proto-oncogenes or amplifying the expression of tumor suppressor genes by regulating the conditions of TFs. Targeting the TFs or applying the inhibitors might a new treatment strategy for lots of BCa patients.

We do acknowledge the limitations in this research, some BCa patients with incomplete clinicopathological information might influence the clinical assessment of the results. Then, the identified TFs were verified in cell culture level and small scale clinical BCa samples, and larger scale of clinical samples are necessary to further validate these results, so as to provide basis for clinical prognosis or detection. Finally, although our research constructed that the five TFs combination serve as the potential progression related biomarkers, they do provide many conveniences for BCa progress and prognosis prediction. The influence of these TFs on the invasion ability and proliferation of BCa need further researched, these TFs might provide new therapeutic targets for these BCa patients. It is necessary to further verify the effects of those identified TFs on BCa *in vivo* and *in vitro*.

## Conclusion

In this research, we determined that the co-expression blue module was obviously correlated with these BCa clinical traits using WGCNA. These selected TFs from co-expression modules enriched in different pathways and cell functions are tightly related to the risk factors and progression of BCa. Furthermore, we identified a series of potential prognostic related biomarkers, which may contribute to the prognosis and treatment of BCa. The five-TFs combination was an independent prognosis biomarker for BCa, and might serve as a potential therapeutic target for BCa patients.

## Supplementary Material

Supplementary table 1.

Supplementary table 2.

Supplementary table 3.

Supplementary table 4.

## Figures and Tables

**Table 1:** Number of TFs in co-expression modules.

Module colors				TFs frequency
Blue				174
Brown				93
Grey				648
Turquoise				166
Yellow				78

**Table 2:** Clinicopathologic characteristics of bladder cancer patients (n=401).

Variables	Total	Risk score		X2	P value
		Low	High		
Age(years)				1.9181	0.1661
>60	294	154	140		
≤60	107	47	60		
Gender				5.5847	0.01812*
Male	297	138	159		
Female	104	63	41		
Tumor_stage				17.456	2.94E-05*
stage I&II	131	44	87		
stage III&IV	270	157	113		

**Table 3:** Univariate and multivariate analysis of clinicopathological factors and the five-TFs combination in BC malignant progression

Trainmarker	Univariate analysis				Multivariate analysis			
	HR	Low 95%CI	High 95%CI	P value	HR	Low 95%CI	High 95%CI	P value
Risk	0.577078	0.426534	0.780757	0.000364*	0.672088	0.491866	0.918345	0.012604*
Age	1.913494	1.298151	2.820520	0.001045*	1.778215	1.204204	2.625840	0.003800*
Gender	1.136034	0.818468	1.576815	0.445792	1.081515	0.774444	1.510342	0.645600
Tumor_stage	2.220953	1.532706	3.218252	0.000025*	1.992851	1.364668	2.910198	0.000358*

## Abbreviations

CBX7: Chromobox 7
HDAC4: Histone Deacetylase 4
EBF2: EBF Transcription Factor 2
NFATC1: Nuclear Factor of Activated T Cells 1
AKNA: AT-Hook Transcription Factor
WGCNA: Weighted correlation network analysis
TCGA: The Cancer Genome Atlas
TOM: topological overlap matrix
GO: Gene Ontology
KEGG: Kyoto Encyclopedia of Genes and Genomes
BCa: bladder cancer
TFs: transcription factor
OS: overall survival
BLCA: bladder cancer
ATCC: American Type Culture Collection
