# Supplementary material for: Comprehensive analysis of Transcription Factors identified novel prognostic biomarker in human bladder cancer
Source: J Cancer. 2021 Jul 25;12(18):5605–21. doi: 10.7150/jca.58484 (PMC8364643; doi:10.7150/jca.58484)
Supplement: Supplementary file 1 — Supplementary table 1. [file jcav12p5605s1.pdf]

Table S1

Name	primers
GAPDH	GTCTCCTCTGACTTCAACAGCG
	ACCACCCTGTTGCTGTAGCCAA
CBX7	AGGAAGAGAGGTCCGAAACCCA
	AGAAGCAGAGCTTCTCCTTGCC
HDAC4	AGGTGAAGCAGGAGCCCATTGA
	GGTAGTTCCTCAGCTGGTGGAT
EBF2	AGCTCCTCTACAGCAACGGTGT
	AGGAGAACTCGGCACATTTCCG
NFATC1	CACCAAAGTCCTGGAGATCCCA
	TTCTTCCTCCCGATGTCCGTCT
ANKA	CTCTGGCAACAGTGAGGTGGAG
	GGAGAGACTTCACACTGAGGTAC
SMAD4	AGTGTCTGTGTGAATCCATATCACTA
	CCTTTATGTTTCTTAGGATGAAAGCAA
MMP13	AGTTCGGCCACTCCTTAGGT
	CATCGGGAAGCATAAAGTGG
IL-2	TGAGAACCTCAAGCTCTCCAG
	CAGATCCCTGTAGTTCCAAAACGA
IL-5	AGGTGATGGGAACTTGATG
	CAGCATCCCCTTGTGCAG
CYP2E1	TCCAACCTACCCCATGAA
	CCAACACACACACGCTTTC
CCNE1	CACAGTATCCCCAGCAAATCTT
	TACAAGGCAGAAGCAGCAAGTA
CDH1	GGCCTTAGAGGTGGGTGACT
	CAGCAAGAGCAGCAGAATCA

si HDAC4	GAGACCCAAUGCCAAUGC
si NFATC1	AGAGAAUUCGGCUUGCACAGGUCCC
si CBX7	GCT GGT TCT GGG AGT TAA AGG
